# Prevention of sexually transmitted diseases among visually impaired people: educational text validation**^1^**

**DOI:** 10.1590/1518-8345.0906.2775

**Published:** 2016-08-18

**Authors:** Giselly Oseni Barbosa Oliveira, Luana Duarte Wanderley Cavalcante, Lorita Marlena Freitag Pagliuca, Paulo César de Almeida, Cristiana Brasil de Almeida Rebouças

**Affiliations:** 2 PhD, RN, Faculdade de Farmácia, Odontologia e Enfermagem, Universidade Federal do Ceará, Fortaleza, CE, Brazil.; 3 PhD, Full Professor, Faculdade de Farmácia, Odontologia e Enfermagem, Universidade Federal do Ceará, Fortaleza, CE, Brazil.; 4 PhD, Professor, Faculdade de Farmácia, Odontologia e Enfermagem, Universidade Federal do Ceará, Fortaleza, CE, Brazil.

**Keywords:** Visually Impaired Persons, Sexually Transmitted Diseases, Self-Help Devices

## Abstract

**Objective:**

to validate an educational text in the context of Sexually Transmitted Diseases (STD) for visually impaired persons, making it accessible to this population.

**Method:**

a validation study, in a virtual environment. Data collection occurred from May to September 2012 by emailing the subjects, and was composed by seven content experts about STDs. Analysis was based on the considerations of the experts about Objectives, Structure and Presentation, and Relevance.

**Results:**

on the Objectives and Structure and Presentation blocks, 77 (84.6%) and 48 (85.7%) were fully adequate or appropriate, respectively. In the Relevance block, items 3.2 - Allows transfer and generalization of learning, and 3.5 - Portrays aspects needed to clarify the family, showed bad agreement indices of 0.42 and 0.57, respectively. The analysis was followed by reformulating the text according to the relevant suggestions.

**Conclusion:**

the text was validated regarding the content of sexually transmitted diseases. A total of 35 stanzas were removed and nine others included, following the recommendations of the experts.

## Introduction

In the context of sexuality, sex is presented as an important component of a healthy sexual experience. When inserted in this scenario, Sexually Transmitted Diseases (STD) are disorders resulting from non-prevention during sex. Factors such as the lack of information may contribute to the occurrence of STDs.

The lack of knowledge in the context of sexual health can be considered a limiting factor among visually impaired persons, as these people require specific components for access to information, such as the use of technologies. In addition, authors consider that STDs pose a risk to the population with vision, but for the visually impaired persons who are subject to social stigmas, these risks become much larger^1^.

Despite the advances, and with public policies having turned to the pursuit of access to education, health and social life of people with disabilities, little has been observed regarding effective actions to encourage and include their emotional and sexual experiences^2^.

These people should be encouraged to practice healthy sex through education on sexual health including contraception and condom use. As shown in the study, visually impaired people also need guidance, taking advantage of technological innovations and advances in which nurses can create various strategies to boost nursing care to this clientele^3^. The technologies for the visually impaired correspond to specific resources adapted to allow the execution of a particular activity impossible from the absence of vision.

The study identifies technologies used in the learning process such as sponken books, the Amplified Reading System and Thermoform (high-relief), in addition to computing resources such as a Braille printer, screen magnification feature and voice synthesizers, also called screen readers^4^. 

The reduced availability of resources for education in health and risk prevention behaviors related to sexual and reproductive health of these clients has been perceived^5^. It is therefore important to create a technology in this context which is validated by judges on the topic of STDs for the promotion of information and encouraging the use of condoms with the view that a large number of visually impaired people can acquire this health knowledge.

The learning process in health involving this population is possible through creative projects. Research conducted with visually impaired people has shown that the use of rhyming text has proved to be an effective strategy in addressing STDs. In designing the subjects, this technological resource used a simple and easy to understand language, thus promoting learning and comprehension^6^.

In the quest for developing materials that promote the inclusion of information for the visually impaired, expert validation by the use of specific methods is recommended which verifies accessibility and efficiency of the resource to be used^4^. Considering the validation process that is considered necessary, the objective of this study was to validate an educational text in the context of STDs for the visually impaired.

## Methods

This is a validation study, classified as an analysis of process or implementation, as well as results and impact. In addition to information on how the process developed, the verification of the research process to elucidate descriptive information was also carried out. The analysis of results verifies if objectives have been achieved, while impact evaluates if the degree of intervention was effective^7^.

The virtual environment of the Laboratory of Health Communication of the Nursing Department of the Federal University of Ceará was used as the study site. Data collection took place between May and September 2012, through the Internet by email.

Seven content experts were the study subjects. The search for the subjects was in consultation with Lattes Curriculum, and through the indication of selected participants. In this way, the non-probability of intentional sampling was used for the selection of participants. For inclusion in the sample, the title of doctor was required, as well as experience in teaching, research and practice of the profession, and scientific articles published in appropriate thematic journals.

For data collection, an adapted (for appearance and content) and validated instrument developed in a previous study^3^ was used, being adapted for the theme of STDs. The instrument was developed for the content validation of a technology also in the form of a rhyme to the theme of breastfeeding for the visually impaired, and the answers used for technology correction ranged from: (5) fully appropriate, (4) appropriate, (3) not applicable, (2) inadequate and (1) fully inadequate.

The text was composed of 78 stanzas, presenting six verses in each stanza, with the second, fourth and sixth verses presenting the same final sound, or in other words, rhyming. The initial part is an introduction on STDs, a relevant theme to the subject, mentioning the right to be informed about diseases, the importance of the recognition, their transmission in general, the need for prevention and early identification. The following diseases were described in this order: AIDS, Chancroid, Condyloma Acuminata (HPV), Chlamydia and Gonorrhea, Herpes, Lymphogranuloma Venereum, Syphilis, Trichomoniasis, Pelvic Inflammatory Disease and Donovanosis.

During the evaluations, the experts were asked to write corrections they deemed necessary in the text, in addition to the notes on the instrument itself. Thus, by being in possession of the completed instruments and the text with additional suggestions, we began to organize the information. 

Data analysis was based on the considerations expressed by the experts through organizing and processing the scores of the instrument, and then quantitatively analyzed in Excel 2010. The data were organized in tables to better understand the results.

In addition, the suitability of the behavioral representation of the items was calculated. This term refers to the calculated statistical value corresponding to the mathematical average of the item considered by the judges. According to the instruments used, the choices of answers 1, 2, 3, 4 and 5 were grouped as follows: 1 and 2 (score = -1), 3 (score = 0), and 4 and 5 (score = +1), where the response of each judge could vary from -1 to +1. The Concordance index (CI) was defined as |∑ f x i| / 7, where f = frequency, i = -1, 0 or 1 and n = number of judges (n = 7). The closer this ratio is to 1.0, the greater the agreement of an item's relevance. The study validated educational material with expert judges and used this analysis to behavioral verification of the items^8^.

For CI analysis, the following values and their respective classifications were used as reference: insignificant (less than 0.20), minimal (between 0.21 and 0.40), bad (between 0.41 and 0.60), good (between 0.61 and 0.80) and excellent (between 0.81 and 1.0)^9^. The items were considered valid for groups ranked "good" and "excellent." For items with "insignificant" rating, "minimal" and "bad", the necessary corrections were made with respect to the experts' suggestions.

The legal provisions were complied with and all subjects signed the informed consent form (CF). The project was approved by the Ethics Committee of the Federal University of Ceará under protocol 37/12.

## Results

The analyzes are presented as three blocks of items related to the assessment tool: Objectives, Structure and Presentation, and Relevance. The Objectives block observed goals or purposes for using the text. The Structure and Presentation block refers to how the present text involves the overall organization, structure, presentation strategy, coherence and adequacy. Finally, the Relevance Block understood characteristics relating to the degree of significance of the material.

Table 1 shows the levels of agreement between judges according to each block and item.

**Table 1:** CI among experts, according to the content assessment tool. Fortaleza, CE, Brazil, 2012

Items		+1	0	-1	CI^*^
Objectives (n=91) ^†^					
	1.1 Contextualizes the problem	7	-	-	1.00
	1.2 Defines STD^‡^ correctly	6	-	1	0.71
	1.3 There are introductions and conclusions for each STD	7	-	-	1.00
	1.4 Portrays the transmission mode of STDS	7	-	-	1.00
	1.5 Addresses the main symptoms of STDS	7	-	-	1.00
	1.6 Emphasizes the types of treatment for STDS	2	2	3	0.14^§^
	1.7 Has reflective content	7	-	-	1.00
	1.8 Emphasizes the importance of prevention	6	-	1	0.71
	1.9 Encourages behavior change and attitude	4	1	2	0.28
	1.10 Causes reflection on damage caused by STDs	6	1	-	0.85
	1.11 The content motivates dialogue	6	-	1	0.71
	1.12 Clarifies possible doubts on the subject	5	1	1	0.57
	1.13 Emphasizes the importance of the problem	7	-	-	1.00
Structure and Presentation (n=56)					
	2.1 It is suitable for men and women	7	-	-	1.00
	2.2 Presents accurate scientific information	7	-	-	1.00
	2.3 Presents information in clear and understandable terms	7	-	-	1.00
	2.4 Text size is suitable	4	-	3	0.14
	2.5 Logical sequence of content	5	1	1	0.57
	2.6 Addresses the main topics on the prevention of STDs	6	-	1	0.71
	2.7 The language is well structured for audience understanding	6	-	1	0.71
	2.8 Doesn't have discriminatory or prejudiced language	6	1	-	0.85
Relevance (n=42)									
	3.1 Emphasizes the key aspect that needs to be strengthened	6	1	-	0.85
	3.2 Allows transfer and generalization of learning	5	-	2	0.42
	3.3 Clarifies the public issues related to the problem	7	-	-	1.00
	3.4 Encourages reflection on the subject	7	-	-	1.00
	3.5 Portrays aspects needed to clarify the family	5	1	1	0.57
	3.6 It is appropriate and can be used in distance health education	7	-	-	1.00
Total		162	8	18	

*Concordance index; ^†^n = number of answers; ^‡^Sexually Transmitted Diseases; ^§^0.14 = ( | 2(1) + 2(0) + 3(-1) | ) / 7

The experts answered all items: the first block (Objectives) received 91 responses, the second (Structure and Presentation) 56, and the third (Relevance) 42 answers. We observed a tendency for experts to opt for the answers in agreement. It was also observed that the majority of responses were adequate or fully adequate (162). Of the 27 items, only 18 had inappropriate or fully inappropriate scores.

On the Objectives and Structure and Presentation blocks, 77 (84.6%) and 48 (85.7%) were fully adequate or appropriate, respectively. Finally, 37 (88.1%) of the responses for the text's Relevance block were adequate or fully adequate. Therefore, the text was valid regarding the proposed objectives, structure and presentation, and relevance.

As shown in Table 1, only seven items of the instrument and its operational definitions did not hold agreement. Despite the fully adequate or appropriate assessment regarding some items, all experts identified in the text some relevant corrections to various aspects of information.

In the Objectives block, item 1.6 - emphasized the types of STD treatment obtained rated as an insignificant index (0.14). In this item, the treatments were then added, corrected or emphasized, corresponding to requests from experts. In relation to item 1.9, two experts appointed it unsatisfactory, for one it did not apply, and the remaining voted it as satisfactory, corresponding to the minimal rating index of 0.28. An expert justified his dissatisfaction by commenting that the change of attitude was not evident on the back, but rather the information was an issue, while two experts believed that it was not relevant to assess this, but they did not justify it in relation to their answer choice.

For item 1.12 - Clarifies possible doubts on the subject, an expert said it was unsatisfactory, another believed the item was not applicable, while the others agreed it was satisfactory. The item had a bad index of 0.57, and one of the experts considered that the text did not clarify doubts on the subject.

Although aspects related to the introduction, transmission mode of diseases and addresses the main symptoms were rated as good (0.85) and excellent (1.00), corrections were identified for text improvement. For example, for Syphilis a word substitution of "marks" for "spots" was suggested because Syphilitic Roseola has recently been updated to the classification of latent and late Syphilis, and this was a greater emphasis on congenital Syphilis.

An improvement of information as to the existence of visible lesions for HPV was requested, considering that viewing these is not always possible. There was marked controversy about reporting the transmission modes of the disease, and regarding Condyloma, the evaluators addressed the need for correcting the manifestation of the disease between men and women.

The reviewers suggested adding Hepatitis B, because according to one of the specialists it is also a sexually transmitted disease and there needs to be work on its prevention. Finally, an expert called for correction in the location of Chlamydia and Gonorrhea outbreak. The text presented the cervix as the main site, however, these are present in the urethra of men and women, and the endocervix in women.

Although section 2.2 received an excellent rating (1.00) where the experts attested to the scientific consistency of information for the Structure and Presentation block, they all included considerations for corrections. In relation to item 2.4, three experts evaluated it as inappropriate or fully inadequate, while pointing out that the text should be reduced. The submitted agreement was insignificant at 0.14, and identified as the second lowest of the results. Therefore, some diseases were removed because the experts considered them as being without occurrence in clinical practice.

Following the recommendations of the experts, a total of 35 stanzas were removed and nine others included. In addition to the withdrawal of all information about STDs that have no epidemiological impact, other diseases that remained and did not compromise the entire content were also canceled as per the guidance of the experts. Withdrawn diseases were Chancroid, Lymphogranuloma Venereum, Pelvic Inflammatory Disease (PID) and Donovanosis. Another item deleted was the family planning content presented at the end of the text. 

In assessing the item 2.5 - The result of the proposed content is logical, an expert described it as inadequate or fully inappropriate, while another considered that the item does not apply to the text. The obtained concordance index was 0.57, also considered bad. Two experts suggested the reorganization of diseases starting with those that have the most impact (AIDS and Syphilis), followed by viral and bacterial diseases. Two others suggested the division of systemic diseases, ulcers and discharges. Regarding the preliminary guidelines, the text has been reorganized, starting with the most impactful and the association of viral and bacterial diseases. The resulting order was AIDS, Syphilis, HPV, Herpes, Hepatitis B, Chlamydia and Gonorrhea and finally, Trichomoniasis.

In dealing with the main topics related to prevention (item 2.6) and use of structured language in simple language (item 2.7), most experts showed agreement. An expert evaluated these as inappropriate or fully inadequate, and requested some terms be replaced to facilitate the understanding of "obtain," "sexual abstinence" and "elephantiasis." The amendments were accepted and these were replaced or removed.

In the Relevance block, items 3.2 - Allows transfer and generalization of learning, and 3.5 - portrays aspects needed to clarify the family, showed bad concordant indices of 0.42 and 0.57, respectively. For item 3.2, two experts considered that it was not possible to transmit and generalize learning for different contexts such as residential, employment and education. As for clarification for the family (young people, adults and seniors) the fourth expert did not perceive the need to reflect the different ages, but only adults and young adults. Another expert believed that regardless of the family, the use of information providing a resolute or enlightening consequence depending on the motive for which it was sought.

## Discussion

In accordance with what was found, seven items of the instrument and its operational definitions did not show agreement, namely: types of STD treatment; encouraging changes in behavior and attitude; clarification of possible doubts on the subject; text size; the content sequence; transfer and generalization of learning and presence of the aspects needed to clarify to the family. Other identified problems were corrections about the characteristics of diseases, adequacy of terms and reinforcing prevention.

Among various health problems is the security of sexual health for the visually impaired. The necessity of preventing STDs by encouraging condom use reinforces the relevance of effective actions that address the importance of controlling these diseases in order to make it known to the population, in addition to strengthening adherence to its use. The production of educational materials can correspond to caring for their needs.

We emphasize the need to validate a technology to contribute to the reduction of risk behaviors of the visually impaired. It starts from the perspective of including people with visual impairment in the context of their sexuality in health education activities, as well as during nursing visits.

For preparing and using educational material on health, how to introduce and present the information can be as decisive as the motivation in continuing to use it, and therefore learning. It is necessary to emphasize the development of educational methods to promote learning, learning transfer, organizational knowledge and excellence^10^. Regarding the prevention of STDs for the visually impaired, it is necessary to consider the motivational impact this can promote depending on how it is introduced, which can be decisive for the user in continuing to use the material.

With the suggestions made by the specialists in respect of the symptoms of the diseases, it was possible to clarify and correct information. In relation to the onset of symptoms in the characterization of Syphilis, study addressed that syphilis rose spots are presented as rounded erythematous macules initially appearing in the secondary stage of Syphilis^11^. Conforming to the analysis, the word used in the text is now "marks" instead of "spots," a term easily understood that corresponds to an injury without necessarily characterizing it.

Following the recommendations of the experts, the literature confirms current Syphilis classification, divided according to the degree of manifestation: recent, latent or late. Recent research is defined as Syphilis of less than one year's duration with the expression of a lesion called chancre, and is composed of the primary and secondary stages; latent Syphilis is known as clinical silence phase, between one to two years after exposure; and late Syphilis can start at the end of the latent phase, and extend for several years^12^.

Considering Syphilis, when there is no one correct approach, vertical transmission results in higher incidence of miscarriages and occurrences of congenital diseases. Prenatal care is a key strategy to reduce the incidence of congenital Syphilis and transmission of HIV from mother to child, with early diagnosis and appropriate treatment^13^. Thus, the addition of a verse emphasizing prenatal care was needed. It is known that the only way to prevent congenital Syphilis is early diagnosis in pregnant women.

As for Condyloma Acuminata, a disease caused by the HPV virus, the text mentions that originated lesions are visible and characteristics are exemplified by the appearance of a cauliflower type wart. A study that evaluated the prevalence of intraepithelial lesions caused by HPV was considered, among other factors, as to the existence of visible lesions to complete the diagnosis^14^. At the request of the experts and based on the literature, corrections were made.

As also observed by experts, the author shows that the majority of infections caused by HPV are asymptomatic or subclinical in both sexes. This means that men can have the same range of manifestations in terms of lesions as seen in women, and small lesions that rapidly progress in a matter of weeks are often detected in clinical practice, even in healthy young people, especially in recent contamination and together with other STDs^15^. Thus, the explanation for the occurrence in both sexes refers to the issue of equal responsibility for both men and women in the prevention of not only HPV, but STDs in general. 

A study conducted on a profile of male users of a STD referral service reported that the adequate late treatment of HPV is a main concern, where it can result in cervical and penile cancer if not treated properly^16^.

With regards to the approach of Hepatitis B recommended in the text, and according to the literature, viral Hepatitis is the major cause of liver disease and Hepatitis B and C can potentially evolve into chronic Hepatitis, Cirrhosis and Hepatocellular Carcinoma which increases mortality from chronic liver diseases^17^.

For the treatment of STDs, the authors state that there currently is no capable cure for genital herpes and that some antiviral drugs are able to decrease the time of the disease and prevent the onset of lesions. Furthermore, daily therapy by symptomatic patients may reduce the risk of transmission to their sexual partner^18^.

We could see the relevance of the information presented in the text and that their corrections were relevant. Therefore, it follows that education aimed at the issue of STDs should be a constant reality for the population, with the goal of people reflecting and understanding that prevention is the main way to control these diseases and minimize their impact^19^.

In Brazil, STDs are considered a serious public health problem. Unfortunately many health centers throughout the country lack appropriately trained medical and nursing staff to assist patients with these diseases^20^. For prevention, condom use is highlighted in the first stanza, as suggested by the experts, and emphasizes the paramount importance of safe sexual practice.

Considering Herpes and supporting the suggestion of the first specialist, studies show that lesions may be present in the genital area of ​​men and women which are not covered by the condom, and thus the condom can only prevent transmission of genital herpes when it covers the whole area infected. Preventive measures include health education for the population, use of barrier methods and chronic suppression therapy^18^.

In addition to the prevention highlighted in the text, recent studies have been conducted in seeking to discuss and verify the impact that the vaccine for women can have on the prevention of HPV^21^ ^-^ ^22^. For Hepatitis B, research shows that this vaccine has proved to be an important factor in the prevention of infection, and is being included in immunization programs^17^.

To find out about STDs and their particularities, it is thought that the knowledge provided and the fear itself of these diseases is enough to be able to encourage change in behavior and attitude. As such, this information should be accessible to the needs of the visually impaired persons.

Health information on the topic of STDs as well as other aspects about themis an effective means to reach the population so that they become aware and active in making decisions about their health needs. For example, inappropriate behavior can be modified so that one is conscious of the problems that may result. One can see that a lack of knowledge is also responsible for the lack of awareness about what is needed. Thus, it is thought that information should be able to encourage behavior change and promote health.

Research shows that even in relation to HIV/AIDS, factors such as low perception of risk or lack of knowledge about the severity of clinical symptoms, among others, limits the search and use of health services, which contributes to late diagnosis of the disease^23^. Thus, in order to raise awareness of the existence and occurrence of STDs, the opportunity for reflection by individuals and prevention must be offered; and in the event of a risky/dangerous situation, awareness about seeking out a health service. Therefore, the use of suitable, attractive and available resources is required.

The size of the educational material is a factor that may help or hinder its use. It is necessary to limit the size of the educational material, because the smaller and more concise it is, the more accessible it will be to the public. The size should be sufficient enough to mention the necessary and essential information. Pages in large numbers with lots of information may discourage health learning^10^. Reducing the material was one aspect corrected to match the request of the experts, by choosing the most prevalent STDs.

Over 30 different types of viruses, bacteria and parasites are sexually transmitted, with the most common STD being chancroid, genital herpes, genital warts, human immunodeficiency virus (HIV), hepatitis B, gonorrhea, chlamydia, syphilis and trichomoniasis^20^. A study by Community Health Agents (CHA) cites Donovanosis and Lymphogranuloma as STDs, which are little known and identified^24^. Chancroid was not kept in the text and Hepatitis B was added as per the expert advice on the composition of the technology.

## Conclusion

The text was validated regarding the content of STDs as being considered an adequate technology by the specialists/experts For analysis of the three blocks of items related to the assessment tool, Objectives, Structure and Presentation, and Relevance were valid when considering that over 80% of the response options were fully adequate or appropriate. The calculation of the majority of the concordance indices of the items received good and excellent ratings, and those that had insignificant, minimal or bad ratings have been corrected.

A total of 35 stanzas were removed and nine others included, following the recommendations of the experts. In addition to the withdrawal of all information about STDs that have no epidemiological impact, other diseases that remained and did not compromise the entire content were also canceled as per the guidance of the experts.

All content experts made relevant suggestions for text improvement. As has been found from the proposed considerations, the problems identified were corrections about the characteristics of diseases, adequacy of terms, identification of types of treatment, reducing the amount of content in the text, displaying the sequence of diseases and enforcing prevention.

Health policies such as STD prevention campaigns are directed to the seeing public, and are not being adapted to those with disabilities, which enhances their vulnerability and leaves them at a disadvantage. The use of computers and the internet facilitate access to educational materials written for this clientele.

In addition to mediating the relationship of the visually impaired with the health service, nurses should be responsible for the effectiveness of care. Health education through adequate resources is a means by which these professionals can ensure quality health care, which can lead to a more inclusive society.

It is believed that this study can contribute to health professionals to becoming more sensitive to the issues of STDs in the context of the visually impaired persons, and new educational technologies can be developed and used. With the encouragement of condom use discussed at various times in the text and the knowledge provided about the occurrence of STDs, this will inspire awareness among the visually impaired persons about the need for prevention and the adoption of safe sexual practices.

A limitation of this study was in the difficulty in identifying experts corresponding to the inclusion criteria, as well as their limited availabilty, which hindered being able to get 7 participating expert subjects. 
